# Fucoidan in Pharmaceutical Formulations: A Comprehensive Review for Smart Drug Delivery Systems

**DOI:** 10.3390/md21020112

**Published:** 2023-02-04

**Authors:** Yusuf A. Haggag, Abeer A. Abd Elrahman, Roland Ulber, Ahmed Zayed

**Affiliations:** 1 Department of Pharmaceutical Technology, Faculty of Pharmacy, Tanta University, El-Geish Street, Tanta 31527, Egypt; 2 Department of Pharmaceutical Sciences and the Biointerfaces Institute, University of Michigan, Ann Arbor, MI 48109, USA; 3 Institute of Bioprocess Engineering, Rheinland-Pfälzische Technische Universität Kaiserslautern-Landau, Gottlieb-Daimler-Street 49, 67663 Kaiserslautern, Germany; 4 Department of Pharmacognosy, Faculty of Pharmacy, Tanta University, El-Guish Street, Tanta 31527, Egypt

**Keywords:** drug delivery, formulation, pharmaceutical industry, fucoidan, nanotechnology

## Abstract

Fucoidan is a heterogeneous group of polysaccharides isolated from marine organisms, including brown algae and marine invertebrates. The physicochemical characteristics and potential bioactivities of fucoidan have attracted substantial interest in pharmaceutical industries in the past few decades. These polysaccharides are characterized by possessing sulfate ester groups that impart negatively charged surfaces, low/high molecular weight, and water solubility. In addition, various promising bioactivities have been reported, such as antitumor, immunomodulatory, and antiviral effects. Hence, the formulation of fucoidan has been investigated in the past few years in diverse pharmaceutical dosage forms to be able to reach their site of action effectively. Moreover, they can act as carriers for various drugs in value-added drug delivery systems. The current work highlights the attractive biopharmaceutical properties of fucoidan being formulated in oral, inhalable, topical, injectable, and other advanced formulations treating life-quality-affecting diseases. Therefore, the present work points out the current status of fucoidan pharmaceutical formulations for future research transferring their application from in vitro and in vivo studies to clinical application and market availability.

## 1. Introduction

Fucoidan encompasses a heterogenous class of polysaccharides found in the extracellular matrix and cell wall of brown seaweeds (Phaeophyta) and marine invertebrates with potential physiological functions. It acts as cellulose and hemicellulose cross-linkers, playing a crucial role in cell wall integrity, in addition to preventing algae dryness during summer and low tide periods, among others [1,2]. Chemically, fucoidan is composed of a sulfated backbone of diverse sugar monomers, mainly L-fucose, as well as galactose, glucose, xylose, mannose, and uronic acids. However, some proteins and minerals such as calcium, magnesium, manganese, copper, potassium, selenium, sodium, and zinc can be also found [3]. The heterogeneity in fucoidan’s chemical composition regarding monomeric composition, glycosidic linkages, sulfation pattern and content, presence of other constituents, and molecular weight has been investigated extensively in the past few years. These variations are mostly associated with different factors, including algae species, season of harvesting, extraction processes, geographical origin, and vegetative phase [4,5,6,7]. The diversity in fucoidan’s biochemical composition has enriched various scientific fields with investigations of potential bioactivities and applications. For instance, fucoidan either with low, intermediate, or high molecular weight has gained much interest recently due to its promising biological and pharmacological properties such as heparin-like anticoagulant, antitumor, anti-angiogenic, anti-inflammatory, anti-hyperglycemic, antiviral, and immunomodulatory bioactivities [8,9,10,11]. As a result, fucoidan-containing products are consumed widely for nutraceutical and health-promoting benefits based on unique molecular mechanisms. Liu et al. reported that fucoidan can be beneficial as a nutraceutical product against obesity and abnormal lipid accumulation within cells through regulating cellular glucose consumption as it reduces glycerol-3-phosphate dehydrogenase (GPDH) functionality. Furthermore, high-molecular-weight fucoidan extracted from *Fucus vesiculosus* has also been found to have antioxidant, antihyperglycemic, anti-inflammatory, and anticoagulant effects which are attributed to multiple mechanisms, including the inhibition of dipeptidyl peptidase-IV enzyme as one of the possible mechanisms involved in the anti-hyperglycemic activity of fucoidan, and its selective inhibition of the cyclooxygenase-2 (COX-2) enzyme, responsible for its anti-inflammatory effect [12]. Other reported mechanisms include the role of fucoidan in modulating oxidative stress and gut microbiota [13,14]. Further investigations have confirmed this activity in perfluorooctanoic acid-associated obesity in an animal model [15]. These fucoidan products used the dried fucoidan extract derived from *Undaria pinnatifida* (a type of brown seaweed) for the preparation of functional foods in the form of cookies and noodles [16]. Edible films are another formulation technique for incorporating fucoidan into alginate-chitosan gel films as nutraceuticals with antioxidant and anti-inflammatory effects [17]. Other cosmeceutical applications (e.g., anti-photoaging and skin protective activity) were shown for fucoidan products against UVB irradiation via its antioxidant effect and its inhibition of oxidative stress markers and matrix metallopeptidase-9 (MMP-9) expressions [18].

**Figure 1:**
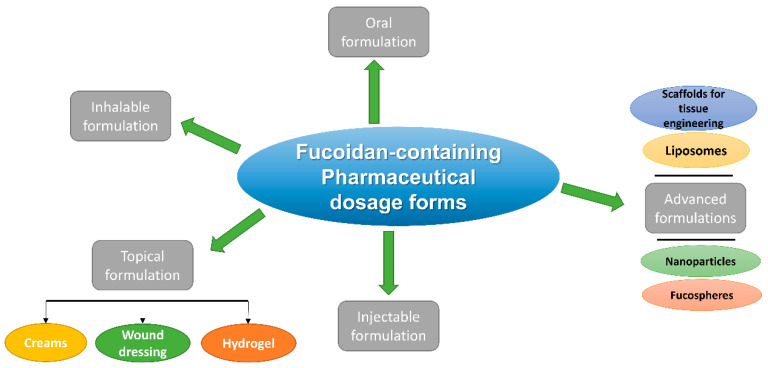
A summary of recently developed pharmaceutical formulations containing fucoidan.

Fucoidan also offers some attractive physical properties enabling its use in diverse pharmaceutical formulation techniques (Figure 1). These physical characteristics include, for instance, mucoadhesion, pH, temperature, and enzyme response. In addition, fucoidan has a strong ability to bind to numerous compounds and macromolecules. The binding affinity is mainly resulting from the negatively charged surface, the degree of sulfation, and molecular weight. Additionally, fucoidan has low apparent viscosity with a pseudoplastic flow, preventing its use as a gelling or thickening agent. On the other hand, upon mixing fucoidan with oppositely charged polymers, gel, matrices, and films can be formed. Moreover, fucoidan is known for its high stability under acidic and alkaline conditions [19].

In the past few decades, considerable progress has been achieved in designing suitable formulations of fucoidan as a therapeutic agent, polymeric drug carrier, excipient, and matrix-forming system. However, there is still a need for additional well-established clinical investigations to evaluate its safety and efficacy on humans.

To write down this review, we used the following keywords in our literature search: fucoidan formulations, drug delivery, fucoidan dosage forms, biological and pharmacological properties of fucoidan, scaling-up marine drugs, and advanced formulations. We included articles related to pharmaceutical formulation and pharmacological properties of fucoidan and excluded articles discussing marine drugs other than fucoidan. The PubMed database was used with a time frame of publications indexed at the time period between 2005 and 2022. The limitations of our review are reported pharmaceutical formulation of fucoidan, their possible dosage forms, advanced drug delivery systems, biological activity, and pharmaceutical applications. We highlight the latest advances in biomedical and pharmaceutical applications of fucoidan as a carrier for drug delivery and tissue engineering scaffolds or as a therapeutic agent on its own. Firstly, the established biopharmaceutical properties of fucoidan such as mucoadhesion, pH, thermal, and enzymatic response, as well as targeting ability, are discussed. Then, the possible dosage forms, routes of administration, and advanced formulation approaches are explained. Finally, the possibilities and challenges of scaling up fucoidan-based products are investigated.

## 2. Biopharmaceutical Properties of Fucoidan

**Figure 2:**
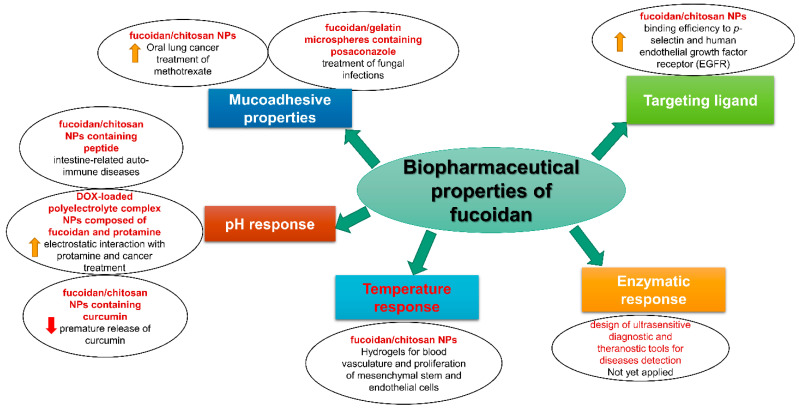
The potential biopharmaceutical properties of fucoidan and their successful uses in pharmaceutical formulations and disease treatment.

In contrast with other polymers, fucoidan exhibits potentially desirable biopharmaceutical properties. Such properties have facilitated fucoidan’s involvement recently in pharmaceutical formulations for more effective disease treatment. These biopharmaceutical properties are discussed in the following subsections and are summarized in Figure 2. 

### 2.1. Mucoadhesive Properties

Mucoadhesive pharmaceutical formulations are designed to ensure intimate contact between the formulation and the human mucosal membranes and the extended release of the drug in the desirable tissue. The mucoadhesion mechanism occurs in three successive stages. Firstly, the mucoadhesive polymer becomes wet and swollen by engulfing an aqueous proportion from the surrounding environment. Secondly, the swollen polymer exposes more relaxed polymer chains. Lastly, these relaxed polymer chains form van der Waals attractions or hydrogen bonds with mucin protein and the mucus layer. Mucoadhesive drug delivery systems can be used for targeting different mucus membranes in the body (e.g., conjunctiva, nasal cavity, buccal cavity, esophagus, upper and lower gastrointestinal tract, rectum, and vagina) [20]. Based on the mucoadhesive properties of fucoidan when mixed with chitosan at pH above 6 to allow the interaction with the acid glycoproteins of mucus (as fucoidan itself does not have mucoadhesive properties), some researchers started to prepare mucoadhesive fucoidan-containing formulations [21].

Recently, researchers have studied the potential mucoadhesive properties of fucoidan/chitosan nanoparticles for the treatment of lung cancer via oral delivery using fucoidan obtained from *F. vesiculosus* with molecular weight (M.W)= 20–190 kDa, fucose content of 44.1%, and sulfate content of 26.3%. These nanoparticles were found to be mucoadhesive to enhance the oral bioavailability of methotrexate as a chemotherapeutic drug. Methotrexate (MTX)-loaded fucoidan/chitosan nanoparticles were 7-fold more effective in inhibiting lung cancer cell proliferation compared to the free drug [12]. Szekalska et al. prepared single-step spray-dried fucoidan/gelatin microspheres containing posaconazole (class II BCS) for the treatment of fungal infections using fucoidan isolated from *Laminaria japonica* with M.W of 10.5 kDa [22]. These posaconazole-loaded fucoidan/gelatin microspheres were tested in simulated vaginal fluid at a pH of 4.2. The results showed that the presence of gelatin increased swelling ability in FUC/GEL microspheres compared to bare fucospheres. However, mucoadhesion studies showed that increasing fucoidan concentration leads to increased detachment force and work of adhesion, with the formulation composed of 0.75% FUC and 2% chitosan giving the highest adhesion values [22].

### 2.2. pH Response 

Among all the explored advanced drug delivery systems (DDS), stimuli-responsive DDS is considered a promising strategy for delivering a drug right to the site of action through targeted delivery. Stimuli applied in this form of drug delivery may be endogenous or exogenous triggers. Endogenous triggers depend on physiological differences between unhealthy and normal tissue. These physiological differences include pH, hypoxia, redox state, enzymes, and regional difference in pressure. On the other side, exogenous triggers include light, magnetic field, ultrasound waves, and electrical and mechanical stimuli. The pH sensitivity is one of the promising and easily applied stimuli-based drug delivery approaches. The physiological pH varies along the gastrointestinal tract and, most importantly, it varies distinctly between healthy cells and tumor cells. Tumor cells possess acidic pH compared to normal cells due to their poor lymphatic drainage and high accumulation of waste product [23,24]. For any polymer to have a pH response, it should have ionizable functional groups interacting with the surrounding medium. Fucoidan is reported to have pH sensitivity owing to its high total number of negatively charged sulfonic acidic groups. However, for pH-responsive tumor targeting, it is crucial to incorporate a basic positively charged polymer (i.e., chitosan) that undergoes ionization upon encountering the tumor’s acidic micro-environment, leading to repulsion among similar charges freeing the core drug content [21]. 

Chitosan itself has poor aqueous solubility at physiological pH. Therefore, to achieve an optimum pH-sensitive polymer coat composition, chitosan is incorporated with fucoidan, taking advantage of its anti-tumor, antioxidant, and immunomodulatory effects. It is important to note that a pH-responsive formula targeted to the tumor site should be injected intravenously to prevent premature release of the drug in the acidic environment of the stomach [25,26]. 

Recently, researchers used an immunomodulatory soluble eggshell protein loaded into fucoidan/chitosan NPs for the management of intestine-related auto-immune diseases using fucoidan isolated from *Laminaria japonica* with M.W of 192 kDa [27]. This study aimed to deliver a peptide therapeutic agent into the intestine, avoiding degradation by gastric enzymes as well as increasing its intestinal absorption. The prepared pH-sensitive chitosan/fucoidan NPs were designed to protect against protein instability, provide sustained release, give synergistic immunomodulatory effects, and enhance the intestinal absorption of the immunomodulator protein [27]. Lu et al. prepared multi-stimuli-responsive DOX-loaded polyelectrolyte complex NPs composed of fucoidan extracted from *Laminaria japonica* (M.W = 80 kDa and sulfate ester content = 34%) and positively charged protamine [28]. This complex proved to provide stability and a long circulation half-life within the blood (pH = 7.4). In this study, the presence of fucoidan enabled electrostatic interaction with protamine, forming a pH-responsive polymer coat. This coat acted as a targeting ligand for p-selectin-expressing metastatic cancer cells. Meanwhile, protamine, as a source of amino acids, can trigger enzyme-responsive drug release. Cancer cells bear protein-digestive enzymes (i.e., trypsin) which hydrolyzed the peptide chains of protamine, leading to the volume extension of NPs with subsequent DOX release [28].

Oral sustained-release fucoidan–chitosan NPs were prepared via the ionic gelation method with a high encapsulation efficiency of curcumin as an anti-tumor drug [29]. The pH-responsive polymer carrier prevented the premature release of curcumin at pH 1.2, while the release was significantly increased upon increasing the pH value to 7. Moreover, at pH = 7, chitosan can enhance the opening of the intestinal tight junctions and thus facilitate paracellular transport of the drug, despite its hydrophobic nature [29].

### 2.3. Temperature Response

Most natural polysaccharides exhibit thermo-responsive properties on their own or after synthetic modification. Many thermo-responsive polymers can respond to thermal change differently. Some polymers have a physical characteristic called lower critical solution temperature (LCST), while others have upper critical solution temperature (UCST). The difference here is that polymers with LCST are present in the form of a solution at room temperature and form chemical cross-linking upon increasing temperature (physiological temperature). On the contrary, polymers with UCST liquefy at high physiological temperatures and form gels below it. Polymers with a transition temperature (~20–40 °C) resembling room and physiological temperature are of immense importance in the biomedical field. This behavior enables researchers to utilize thermo-responsive hydrogels in various biomedical applications, including “smart” stimuli-responsive drug delivery systems, tissue engineering scaffolds (regenerative medicine), and gene therapy [30,31].

Fucoidan has a non-gelling nature due to its low viscosity. The rheological behavior of fucoidan is greatly dependent on multiple variables such as seaweed species, molecular weight, number of sulfate groups, and uronic acid positions [3]. Unfortunately, no studies have reported whether fucoidan itself exhibits upper or lower critical solution temperature. However, previous studies indicated that fucoidan, when mixed with other natural polysaccharides, can form synergistic interaction either via hydrogen bonds, van der Waals forces, and electrostatic interactions by the presence of charged sulfate and hydroxyl groups. Analogously, xanthan polysaccharides cannot form a gel on their own, but gel formation occurs after its synergistic incorporation with another gelling agent such as gelatin and glucomannan [32]. For instance, chitosan and other gel-forming materials are incorporated with fucoidan to form a hydrogel formulation. 

Researchers used this idea to prepare a hydrogel for improving the proliferation of mesenchymal stem cells and endothelial cells. The study aimed to use fucoidan hydrogel for stimulating blood vasculature for engineered tissues [33]. The researchers prepared stromal cell-derived factor-1 (SDF-1)/vascular endothelial growth factor (VEGF) encapsulated in chitosan/fucoidan nanoparticles. The aforementioned nanoparticles were impeded into a hydrogel-forming matrix composed of chitosan, gelatin, and *β*-glycerophosphate. The gelling temperature was 35 °C, suggesting the convenient injection process of the formulation, while the hydrogel phase was formed only after entering the body [33]. It is worthwhile to note that chitosan polymer does not show thermo-response unless mixed with *β*-glycerophosphate solution, and it forms H-bonds with fucoidan in a temperature above 35 °C [34,35]. 

### 2.4. Enzymatic Response

Fucoidan is a non-starchy polysaccharide at which the sugar monomers composed mainly of fucose are linked via α-(1,3) glycosidic linkage. Nevertheless, some other glycosidic linkages occasionally contribute, such as α-(1,4), α-(1,2), or α-(1,6). In addition, trace amounts of galactose, xylose, and glucuronic acid have been reported, and hence, it shows little to no degradation by digestive glycosidase enzymes [36]. The presence of glycosidic bonds in polysaccharides makes them labile to degradation by enzymes such as hyaluronidase and matrix metalloproteinases (MMPs), which act on the dissociation of the cells’ extracellular matrix [37,38]. On the other hand, like most polysaccharides, fucoidan may be labile to degradation by some other enzymes and in analogy to its interaction with MMPs abundant at tumor sites, where fucoidan is reported to inhibit their over-expression [39]. However, this theory needs further investigation in the future to determine whether fucoidan could show an enzymatic response that could be exploited in site-specific drug targeting based on the biocatalytic action of enzymes [37]. Furthermore, the enzymatic response not only facilitates targeted drug delivery but also enables the design of ultrasensitive diagnostic and theranostic tools for the detection of these diseases [40,41]. Unfortunately, no published studies have tried to use the enzyme-responsive properties for targeting different drugs in cancerous tissues. 

### 2.5. Targeting Ligand

Ligand-mediated endocytosis is a promising mechanism for achieving targeted drug delivery. The endocytosis process is important to concentrate the highest possible effective concentration of chemotherapeutic agent into cancer cells, avoiding its cytotoxic adverse effect on normal cells [30]. Previous studies have reported that multiple sulfated oligosaccharides and polysaccharides (i.e., fucoidan, heparin, and dextran sulfate) can effectively bind to lectin receptors such as p-selectin [42]. The p-selectin receptor is overexpressed on activated platelets and is responsible for leukocyte rolling and trapping on platelet aggregates and the extravasation process. Additionally, p-selectin is found to be overexpressed by tumor cells and is responsible for promoting the adhesion process of cancer cells to endothelium and hence facilitating metastasis [43]. Liu et al. prepared chitosan/fucoidan NPs using fucoidan extracted from *F. vesiculosus* and showed their high binding efficiency to p-selectin and human endothelial growth factor receptor EGFR [44]. Rouzet et al. prepared radiolabeled (Technetium ^99m^Tc) LMW fucoidan with the composition of L-fucose 45%, D-glucuronic acid 25%, and 27% ester sulfate for in vivo imaging to detect platelet-rich arterial thrombi and acute myocardial ischemic events [45]. The idea of this research was based on the high affinity of fucoidan to p-selectin, which was found to be 10,000 times greater than other polysaccharides with the lowest possible off-target binding [45]. Novoyatleva et al. utilized fucoidan as a targeting therapeutic agent for pulmonary arterial hypertension-related hypoxia that is associated with p-selectin overexpression and upregulation [46]. Based on fucoidan/p-selectin affinity, fucoidan significantly reduced pH-induced hypoxia in mice, detected by restoring right ventricular performance and decreased vascular pulmonary remodeling [46].

## 3. Pharmaceutical Dosage Forms of Fucoidan and Their Different Routes of Administration

**Figure 3:**
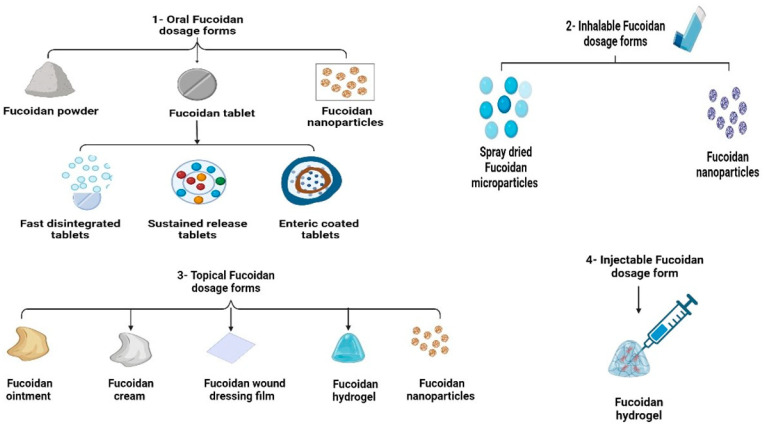
Different dosage forms and pharmaceutical formulations containing fucoidan.

Fucoidan-containing pharmaceutical formulations are classified and summarized in Figure 3 based on the route of administration. In addition, they are discussed in detail in the following subsections. Previous literature reported that fucoidan exerts many interesting biological, pharmacological, and antimicrobial characteristics; for instance, Krylova et al. studied the antiviral activity of naïve and an enzymatically modified fucoidan derivative of high molecular weight isolated from *Fucus evanescens* against herpes viruses (HSV-1, HSV-2), and the results showed that the introduction of both types to the tested cells in vitro produced the highest antiviral activity as a protective effect before infection, while the in vivo tests using HSV-2 vaginally infected mice treated with intraperitoneal fucoidan at a dose of 10 mg/kg/d showed a significant reduction in virus replication (HSV-2 titer was decreased by 2 to 3 lg TCID50/mL) and significantly prevented lethal infection outcomes [47]. 

Furthermore, fucoidan from different species is proven effective against different bacterial strains; for instance, fucoidan extracted from *Sargassum polycystum* demonstrated significant inhibition of the in vitro bacterial growth of *E. coli*, *S. aureus*, and *S. mutans*, with the highest inhibitory effect observed with *Pseudomonas aeruginosa* (21 ± 1.0 mm at the concentration of 50 μg/mL). The in vivo tests performed on *P. aeruginosa*-infected zebrafish treated with 15 mg/0.1 kg fucoidan pre- and post-exposure to the pathogen revealed that the fucoidan-pretreated fish showed lower mortality (10%) than fucoidan-post-treated fish (16.6%), while the control group showed total mortality within 20 days [48].

Antifungal activity of fucoidan extracted from *Undaria pinnatifida* was investigated by testing fucoidan against three fungal species, namely *Aspergillus flavus*, *Aspergillus fumigatus*, and *Mucor* species, and showed a larger diameter of inhibition zone for *A. fumigatus* (11.83 ± 1.0 mm) followed by *A. flavus* (8.5 ± 0.87 mm), with *mucor* species showing the least response, indicating its resistance to fucoidan treatment [49]. 

Interestingly, fucoidan as a marine polysaccharide possesses a prebiotic activity on gut microbiota as it can enhance the growth of beneficial gut flora and modulate gut dysbiosis resulting from the transformation of beneficial bacteria into harmful pathogens. Furthermore, fucoidan was reported to modulate cellular immunity, support the intestinal epithelial barrier, and reduce the expression of inflammatory mediators such as TNF-α and IL-6, and it can also directly promote the growth of the beneficial *Lactobacillus* species [50].

Zhu et al. investigated the prebiotic effect of fucoidan (from *Undaria pinnatifida*, M.W= 276 kDa with sulfate content = 29.65%) on *Lactobacillus rhamnosus* (gut probiotic bacteria with antimicrobial effect against intestinal pathogens) and reported that fucoidan enhanced the growth and metabolic functions of probiotics and thus, fucoidan-treated probiotics showed significant antibacterial effects against pathogens such as *E. coli*, *S. aureus*, and *E. faecalis*, detected by an increase in the diameter of the inhibition zone in a concentration-dependent manner [51].

Based on previous studies suggesting that fucoidan could be used as a supplementary therapy for cancer patients to improve their chemotherapeutic response, a double-blind randomized case control study was performed on 54 patients with metastatic colorectal cancer. Tsai et al. studied the effect of low-molecular-weight fucoidan (extracted from *Sargassum hemiphyllum*, M.W 0.8 kDa and fucose content of 210.9 ± 3.3 mmol/g, and sulfate content of 38.9% ± 0.4% (*w/w*)) as a dietary supplement within a period of 11.5 months, and the results showed that the disease control rate was significantly greater (by 23.6%) in the study group compared the control group; the two groups were not significantly different in terms of quality of life and reported adverse effects [52].

### 3.1. Oral Fucoidan Formulations

Although fucoidan has high solubility in water, it has poor gastric solubility and limited absorption from the stomach and upper gastrointestinal tract. In addition, it experiences degradation by normal flora in the lower gastrointestinal tract, producing oligosaccharides and short-chain fatty acids which are rapidly eliminated from the bloodstream by reticuloendothelial clearance. Taking advantage of the poor solubility and low gastric absorption of orally administered fucoidan from the stomach, it can be used as a gastro-protective dietary supplement. Fucoidan can be used orally as a physical barrier and as an anti-inflammatory and oxidative stress suppressor for managing gastric ulcers. In addition to the gastro-protective effect, fucoidan is useful for maintaining the chemical stability of acid-labile drugs in the stomach. In addition, fucoidan can control the release of active pharmaceutical ingredients via pH-sensitive behavior, allowing the release of the drug only in the slightly basic medium of lower GIT [27,53,54]. 

The formulation of fucoidan powder in oral tablet dosage form has some challenges based on fucoidan’s physicochemical properties [38]. For example, fucoidan powder is hygroscopic with low flowability due to its irregular particle surfaces and wide size distribution ranging from 10 to 500 μm. The other formulation challenge is the long disintegration time associated with tablets with high dry powder extract content. Therefore, researchers used some tablet excipients such as sodium croscarmellose, crospovidone, lactose monohydrate, and microcrystalline cellulose to improve the disintegration and flowability, engaging the wet granulation technique [55]. The physical properties of fucoidan powder are reported in Table 1.

Zhao et al. investigated the effect of fucoidan molecular weight on its oral bioavailability and antithrombotic activity in electricity-induced arterial thrombosis in rats. Plasma and urine levels of low-molecular-weight (LMW) and medium-molecular-weight (MMW) fucoidan extracted from *Laminaria japonica* were analyzed. LMW showed higher absorption and better oral bioavailability compared to MMW fucoidan [56]. 

Oral administration of fucoidan from *Saccharina japonica* has been reported to reduce allergic symptoms (degranulation of mast cells) in a murine model [57]. Oral fucoidan is responsible for inducing the secretion of the immune-modulatory factor (galectin-9) by intestinal epithelial cells. In addition, oral administration showed a significant reduction in mice’s rectal temperature as a sign of allergic reaction compared to intraperitoneal injection [57]. 

As mentioned before, oral delivery of fucoidan is limited by its low solubility in gastric fluid and poor intestinal absorption. This poor absorption was attributed to the negatively charged sulfate ester groups of fucoidan. Therefore, capping these negative charges using an oppositely charged compound or adding an intestinal tight junction opening agent (i.e., berberine) are among the strategies adopted to enhance its oral bioavailability [26,58]. Oral delivery of fucoidan/chitosan-coated MTX nanoparticles was investigated in vitro. The stability of the tested formulations was assessed in a simulated gastric fluid at a lower stomach pH of 1.6 and 2.7. The amino groups (NH_2_^+^) on chitosan and sulfate groups (SO_3_^−^) on fucoidan were ionized, indicating a strong electrostatic interaction between polymers and high encapsulation efficiency of MTX. On the other hand, upon increasing the pH value to 6–7.4, representing the environment in the duodenum, the chitosan amino groups became deprotonated, resulting in the reduction in the electrostatic interaction between the polymers and subsequent drug release [21].

**Table 1:** Physical properties of fucoidan powder.

	Tablets	Spray-Dried Microspheres
Appearance	Brown fine powder [55]	Microspheres [59]
Taste	Bitter [55]	N/A
Solubility	Soluble in water [55]	Soluble in water [59]
Mass loss on drying	≤5% [55]	N/A
Mass moisture gain after 1 day	~4% [55]	N/A
Mass moisture gain after 4 days	~10% [55]	N/A
The number of sulfate groups determined by turbidimetry	≥25% [55]	N/A
Bulk density before compression	0.54 ± 0.06 g/cm^3^ [55]	0.45 ± 0.06 [59]
Bulk density after compression	0.80 ± 0.05 g/cm^3^ [55]	N/A
Tapped density	0.79 ± 0.06 [55]	0.77 ± 0.19 [59]
Compressibility coefficient	0.05 [55]	N/A
Carr index	32.5 ± 0.8% [55]	N/A
Angle of repose	55 ± 1° [55]	N/A
Hausner ratio	1.48 ± 0.07 [55]	1.71 ± 0.12 [59]
Morphology	Irregular particles [55]	Unloaded microparticles showed smooth surfaces while drug-loaded microparticles showed irregular surfaces [60]
Particle diameter	10 to 500 µm [55]	1.62 ± 0.8 µm [58]

Tran et al. developed fucoidan-based oral sustained-release tablets composed of hydroxypropyl methylcellulose (HPMC) and polyethylene oxide (PEO) as a hydrophilic release rate-controlling polymer matrix [61]. The fucoidan release profile from PEO and HPMC-containing tablets revealed a sustained release of fucoidan for 24 h. The PEO polymer was superior to extend the release of fucoidan compared to HPMC-composed tablets [61]. 

Tsai et al. studied a self-assembled nanosystem from trimethyl chitosan (TMC) and fucoidan (from *F. vesiculosus*) for oral delivery of insulin [62]. The selection of TMC was based on its mucoadhesive and intestinal permeation-enhancing effect via paracellular transport owing to its quaternary ammonium groups. Fucoidan was selected due to its hypoglycemic effect, attributed to the inhibition of α-amylase and α-glucosidase activity and induction of insulin secretion. The in vitro release study of TMC/fucoidan-coated insulin showed a reduction in insulin release in simulated gastric fluid at pH 2.0. These results were attributed to the shielding effect of polymeric nanoparticles against insulin degradation in gastric fluid. Interestingly, upon replacing TMC with chitosan, the release rate of insulin was faster as chitosan undergoes deprotonation at low pH of the stomach, while TMC bears more positive electrical charges, which increased its stability [62]. 

Da Silva et al. studied the effect of glutaraldehyde cross-linking to circumvent burst drug release on chitosan/fucoidan NPs for oral delivery of fucoidan (extracted from *F. vesiculosus* with M.W = 20–200 kDa) as an antithrombotic agent [63]. The chemical reaction between glutaraldehyde and chitosan yielded imine bond formation. This chemical bond decreased NPs’ dissolution in gastric acidic fluid and improved fucoidan encapsulation efficiency [63].

An enteric-coated fucoidan was formulated for targeted delivery to human colon cancer [64]. Enteric-coated fucoidan tablets were composed of Kollicoat MAE 100P as an enteric film-forming polymer, polyethylene oxide (PEO) as a swellable matrix-forming polymer, and fucoidan as an anticancer agent with citric acid as an acidifier. This unique fucoidan formulation aims to guard against the premature release of fucoidan into the stomach to sustain its release [64]. Further investigations including in vivo absorption and release studies are still required to fully address the efficiency of fucoidan-based oral formulations.

### 3.2. Inhalable Fucoidan Formulations

The pulmonary route of administration has gained much attention as a non-invasive administration route for deep local alveolar delivery of different drugs. Pulmonary drug delivery can also be used for systemic absorption due to the high alveolar absorptive surface area (∼100 m²). This route of administration is characterized by the presence of a thin absorptive layer, rich blood supply, and minimal degradation enzyme activity. Furthermore, pulmonary drug delivery offers the advantages of bypassing proteolytic gastrointestinal degradation and hepatic first-pass metabolism. Therefore, the pulmonary route is more suitable for the systemic delivery of proteins and/or peptides [65]. 

Natural polysaccharides can act as a carrier for micro/nano aerodynamic particles and aerogels intended for pulmonary delivery [66]. The sulfated fucose polysaccharides can be easily docked in the surface receptors of alveolar macrophages, which host mycobacterium tuberculosis (TB). Researchers investigated the use of spray-dried fucoidan microparticles using fucoidan from *Laminaria japonica* as an inhalable formulation containing both isoniazid (INH) and rifabutin (RFB) [60]. This dosage form containing combined therapy for TB improved the patient compliance with treatment due to targeted drug delivery [60]. The aerodynamic properties of this formula were evaluated, and the erratic surface morphology of the microparticles showed favorable flowability and dispersibility, indicated by low tapped density. The aerodynamic diameter of these microparticles was around 2–4 µm, producing favorable deposition into alveoli [59]. 

Huang et al. developed self-assembled fucoidan/chitosan nanoparticles for intra-tracheal instillation of gentamycin to treat pulmonary infections using fucoidan isolated from *F. vesiculosus* [67]. This study aimed to reduce ototoxicity and nephrotoxicity associated with systemic gentamycin administration. This model exhibited a biphasic release pattern where hydrophilic gentamycin particles present on the surface of the nano-system give initial burst release, followed by the extended release of the entrapped drug over 72 h [67]. 

Dutot et al. investigated the immunomodulatory effect of fucoidan solution extracted from *Ascophyllum nodosum* algae. Incubating bronchial epithelial cells with fucoidan solution for 1 h reduced the cytokine/chemokine mRNA expression, while incubating cells with fucoidan for 24 h reduced COX-2 and PGE2 production [68].

### 3.3. Topical Fucoidan Formulations

The topical drug delivery systems include ointments, creams [69], nanogels and hydrogels [70], wound dressings [71], thin films [72], and smart stimuli-responsive systems [73]. There is a growing tendency by the Food and Drug Administration (FDA) to reformulate different drugs such as anti-inflammatories, analgesics, wound healing enhancers, etc., to be in topical dosage forms. The reason for this is to improve these drugs’ efficacy at the site of action while reducing their possible side effects. The incorporation of active pharmaceutical ingredients (APIs) into a carrier for the topical application provides substantial merits, for instance, enhanced transdermal permeation, protection against first-pass metabolism, ease and convenience of administration, non-invasive drug delivery, and localization of therapeutic effects at the target site of action [69,70]. 

Fucoidan, as a fucose-rich polysaccharide, is known to exert anti-inflammatory, immune-modulatory, and heparin-like anticoagulant action. Furthermore, it has been proven to mediate fast skin regeneration and re-epithelialization by enhancing the migration and build-up of fibroblasts. Fucoidan is used for inhibiting enzymes responsible for the hydrolysis of dermal elastic fibers (elastase, tyrosinase, and collagenase) and the suppression of IgE associated with allergic and inflammatory reactions [71,72]. As a result of the relatively high molecular weight, negatively charged sulfate groups, and hydrophilicity, fucoidans generally have a low skin permeation coefficient. It was also found that the anti-inflammatory effect of fucoidan, especially that based on the inhibition of protein denaturation, is dependent on the fucose and sulfate content of the extract obtained from five different brown seaweed species, which are *Saccharina japonica, F. vesiculosus, Fucus distichus, Fucus serratus,* and *Ascophyllum nodosum* [74].

The pharmacokinetic behavior of fucoidan ointment after topical application was studied using carrageenan-induced paw inflammation in a rat model compared to intravenous administration [73]. The tested formulation contained fucoidan, transcutol as a penetration enhancer, and polyethylene glycol (PEG 400) as a surfactant. The plasma levels of topical fucoidan (100 mg/kg) exhibited a longer half-life of 20.75 ± 9.43 h compared to 9.47 ± 2.34 h after IV administration. This prolonged half-life is attributed to the quick drug penetration and retention of the formula in the form of skin and striated muscle reservoirs [73]. 

The effect of topical application of fucoidan extracted from two different sources (*Undaria pinnatifida* extract, containing 85% fucoidan, and a *F. vesiculosus* co-extract, containing 60% fucoidan and 30% polyphenol) on the skin was evaluated [75]. Both extracts showed inhibition of enzymes responsible for the hydrolysis of dermal elastic fibers (elastase, tyrosinase, and collagenase). In addition, both extracts increased the expression of the human Sirtuin 1SIRT1 protein, counteracting the effect of UV radiation and oxidative stress. Furthermore, both extracts activated Toll-like receptors 2 and 3 with the expression of antimicrobial peptides and wound healing signals by 387% and 229%, respectively [75].

Barbosa et al. prepared methotrexate (MTX)-loaded chitosan/fucoidan nanoparticles as a topical drug delivery system for the management of skin inflammatory conditions using fucoidan extracted from *F. vesiculosus* with M.W 50–190 kDa [76]. This study aimed to avoid the systemic side effects associated with MTX. In vitro skin permeation assay using a pig ear model showed that chitosan/fucoidan NPs increased the drug flux rate and apparent permeability coefficient of MTX, and hence, its anti-inflammatory activity compared to free MTX [76]. A mixture containing fucoidan (0.3% *w/w*) and dexamethasone was topically applied in vivo for mice with induced atopic dermatitis [77]. The fucoidan/dexamethasone-treated group showed markedly less redness and erythema compared to the control group after 2 weeks of topical application [77]. The different topical fucoidan preparations and their rheological properties are summarized in Table 2.

Aside from local transdermal therapeutic effects, fucoidan from *Sargassum* sp. was incorporated into a nano transdermal patch as a bioactive anti-cancer agent against metastatic breast cancer cells, as it interferes with the Bcl-2, Bcl-xL, Bcl-w, and Bad pathways involved in cancer cells’ transformation and malignancy, as well as its chemoprotective effect exemplified by the ability of fucoidan at lower doses to selectively produce apoptosis to cancer cells without causing any toxicity to normal cells in vitro [78].

#### 3.3.1. Fucoidan Creams

Obluchinskaya et al. prepared a fucoidan-based cream with anti-inflammatory action [79]. Formulations contained fucoidan (from *F. vesiculosus* with M.W of 735 kDa), olive oil, hydrogenated castor oil, and a surfactant such as poloxamer 407, geleol, gelucire, lanolin, or cremophor^®^. The highest fucoidan release in vitro was observed with the formulation containing poloxamer 407 as a surfactant. Moreover, poloxamer 407 increased the colloidal stability and enhanced the rheological properties of the formulation. In the same context, the effect of several penetration enhancers, such as dimethyl sulfoxide DMSO, transcutol P, and polysorbate 80, on fucoidan release was assessed. The use of transcutol P increased the diffusion of fucoidan into the agar plate with superior spreadability of the formulation containing transcutol P over polysorbate 80. On the contrary, the formulation containing DMSO showed the slowest release and the poorest spreadability [79].

#### 3.3.2. Fucoidan Wound Dressing Films

Wound dressing films are a simple, low-cost, and non-invasive choice for the management of wounds and promotion of healing. These films should include some major features such as flexibility, mechanical strength, and a physical barrier. These topical films show the ability to absorb wound exudates and evaporate moisture content. Furthermore, wound dressing films can act as a drug delivery system for antibacterial and tissue regeneration promoter genes [80,81,82]. 

Sezer et al. prepared chitosan/fucoidan wound dressing film for dermal burn healing in male New Zealand rabbits using fucoidan (from *F. vesiculosus* with M.W = 80 kDa) [83]. The prepared formulations with varying chitosan/fucoidan ratios were studied concerning water vapor permeability, exudate absorption capacity, mechanical strength, and film thickness. Increasing the concentration of chitosan led to an increase in mechanical strength as well as film thickness. On the other hand, with an increasing concentration of fucoidan, the water absorption capacity was optimized, leading to efficient wound exudates’ absorption by the thin film. The in vivo wound healing study showed wound contraction and scar formation with subsequent re-epithelialization after 14 days of treatment by chitosan/fucoidan wound dressing film [83].

#### 3.3.3. Fucoidan Topical Hydrogels 

Hydrogels are materials that have the ability to absorb water and swell upon embedding in an aqueous environment. Pharmaceutical hydrogels are composed of physically or chemically cross-linked water-insoluble polymers with hydrophilic functional groups and incorporated high water content of 90% *w/w*. Hydrogels provide a variety of physically and biologically interesting characteristics that simulate the physiology of natural tissues. These hydrogels are characterized by softness, flexibility, and a high surface area, along with swelling behavior and high loading capacity of drugs [3,84]. 

Fucoidan is a hydrophilic polysaccharide having some interesting physical characteristics needed for dermal burns and wound treatment. These characteristics are high exudate absorption capacity (high swelling index), mucoadhesion, adequate hygroscopicity, and oxygen permeability. In addition, its pharmacological activity includes heparin-like anti-coagulant, anti-thrombotic, and anti-inflammatory effects [85].

Karami, et al. prepared a sun-protective hydrogel to guard against solar ultraviolet radiation (UVB), composed of silibinin (a silymarin derivative), which inhibits UVB-induced apoptosis and DNA damage [86]. Silibinin has low systemic bioavailability due to its low solubility and instability. Silibinin was loaded into a fucoidan/chitosan hydrogel matrix. The choice of fucoidan was based on its photo-protective effect against UVB. Fucoidan inhibits UVB-induced matrix metalloproteinase 1 (MMP-1) expression in human skin, which is responsible for mediating the degradation of different keratinocyte components. The results showed that UVB-irradiated skin treated with silibinin hydrogel displayed lower oxidative stress and significantly lower levels of H_2_O_2_ compared to untreated irradiated skin [86]. 

Rao et al. developed a polyelectrolyte complex matrix composed of chitosan and fucoidan (from *F. vesiculosus* M.W = 46.4 kDa) as a cell-based therapy for the management of diabetic wounds [87]. In this study, the researchers used the chitosan and fucoidan hydrogel matrix to deliver platelet-rich plasma (PRP) as a source of growth factors and cytokines. The hydrogel formulation helped to overcome the poor shelf-life of PRP and sustain the release of its contained proteins. Sustained release of PRP was obtained for up to 72 h at 37 °C, with in vivo and in vitro studies reporting significant cell proliferation and collagen deposition [87]. 

For achieving wound healing associated with skin cancer conditions, Shanmugapriya et al. prepared a laser-mediated photodynamic therapy system [71]. The formulated hydrogel matrix was composed of fucoidan, chitosan, alginate, carboxymethyl cellulose (CMC), and gellan. Gellan gum was added to maintain hydrogel stability in the presence of metallic ions while CMC was added to enhance the mechanical strength of the hydrogel matrix [88].

### 3.4. Injectable Fucoidan Formulations

Injectable hydrogels should have the ability to undergo a phase transition in response to temperature changes, particularly from ambient temperature to physiological temperature. These systems permit in situ hydrogel injection in a convenient minimally invasive solution form with subsequent solidification inside the body. Hydrogel formation occurs immediately after temperature change without the need for chemical initiators [31]. As previously mentioned, fucoidan cannot form an injectable thermo-responsive gel matrix unless mixed with another thermo-responsive polymer, i.e., chitosan, hyaluronic acid (HA), gelatin, xyloglucan, etc. [89]. 

Lu et al. prepared an intra-articular platelet-rich plasma (PRP) hydrogel system based on low-molecular-weight fucoidan extracted from *Laminaria japonica* [90]. Gel formation was achieved by the incorporation of fucoidan with several gelling agents (i.e., hyaluronic acid, gelatin, genipin) to ensure the formation of a randomly distributed cross-linked gel matrix with high mechanical strength and viscoelasticity. The results of this study showed that fucoidan provided a sustained release of growth factors and enhanced the role of PRP in suppressing inflammatory responses [90]. 

Methacrylated fucoidan was cross-linked with polyvinyl alcohol to form a cell-loaded hydrogel formulation for tissue engineering. The problem reported with previous hydrogel-based formulations is the permeation of inflammatory mediators such as IL-1β and TNF-α, which cause apoptosis in the implanted cells. The addition of fucoidan (from *F. vesiculosusas*), a natural polysaccharide, into PVA hydrogel provided biological functionality as it has cell-binding properties as well as an immune-modulatory effect. The permeation of inflammatory mediators was tested using a side-by-side diffusion chamber, and permeation data revealed minimal diffusion of IL-1β while maintaining the beneficial diffusion of bovine serum albumin [91]

### 3.5. Advanced Fucoidan Formulations

**Figure 4:**
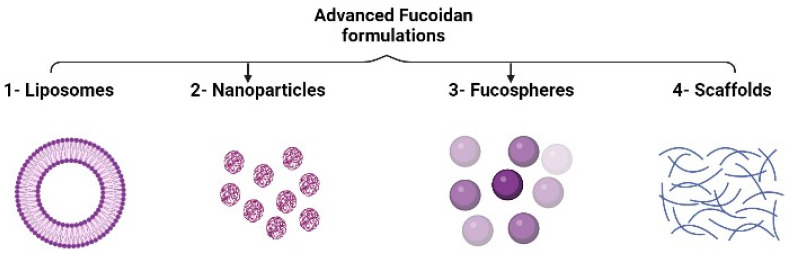
Advanced fucoidan formulation approaches.

Advanced fucoidan formulations include liposomes, nanoparticles, fucospheres, and scaffolds, as illustrated in Figure 4. 

#### 3.5.1. Liposomes 

Liposomes have several beneficial properties, especially in cancer treatment, when compared to other nanosystems. These properties include improving drug solubility, stability, and delivery to specific target sites [92].

Fucoidan extracted from *F. vesiculosus* was encapsulated into a nano-sized liposomal carrier composed of lecithin (phosphatidylcholine) and tested for anticancer and immunomodulatory effects. The results of this study showed increased anticancer activity as well as a reduction in the levels of interleukin-6 and tumor necrosis factor-α compared to fucoidan nanoparticles [93]. 

Salviano et al. synthesized a fucoidan derivative (cholesteryl–fucoidan) loaded with usnic acid as a liposomal drug delivery system for the treatment of *Mycobacterium tuberculosis* infection. Fucoidan-coated liposomes showed significantly lower IC_50_ (8.26 ± 1.11 μM) compared to blank liposomes due to the higher cellular uptake and cellular internalization of the hydrophobic fucoidan derivative liposomal formulation [94]. 

Zhang et al. proposed that liposomes dispersed in a fucoidan matrix can be a promising delivery system for bioactive nutraceutical compounds. Fucoidan can protect drug-loaded liposomes against leakage and burst release and hence improve their overall bioavailability [95].

#### 3.5.2. Nanoparticles

Nanotechnology-based drug carriers are highly prominent in the area of targeted drug delivery for the treatment of various diseases [96,97,98,99]. Targeted delivery of chemotherapeutics is greatly beneficial because these drugs suffer from low aqueous solubility, rapid clearance, and high toxicity. All these delivery limitations can be overcome by the use of biocompatible and biodegradable polymeric nanocarriers [100,101,102].

Recently, fucoidan has played a key role in nanotechnology-based medicine for different biomedical applications. Fucoidan in nanomedicine can be used as a nanocarrier for many drugs or it can be combined with different cationic polymers to encapsulate different cargos, besides being used as an effective therapeutic agent on its own [10,103]. 

Fucoidan is a promising carrier for nanoparticle formulation. The formulation of fucoidan nanoparticles by the self-assembly technique enables fucoidan particles to arrange themselves into a capsule structure that is ready for drug entrapment. The ionotropic cross-linking of fucoidan with polymers having opposite net charge (e.g., chitosan, Polyallyamine hydrochloride, Polyethyleneimine, Hexadecylamine, isobutyl cyanoacrylate) is a common technique for the preparation of drug-loaded nanoparticles [25].

Choi et al. synthesized chitosan–fucoidan polymeric nanoparticles via ionic gelation cross-linking as a drug delivery vehicle for Piperlongumine (PL) [104]. The prepared nanoparticles improved the aqueous solubility and bioavailability of (PL) as a pro-oxidant anti-cancer drug. These NPs showed sustained release over 24 h without displaying any burst effects. The results showed a remarkable increase in intracellular ROS (oxidative stress) of the tested cancer cells, indicating that the polymeric carrier system facilitated the intracellular uptake of PL [104]. 

A quaternary ammonium chitosan (QCS)/depolymerized fucoidan nanosystem was prepared and tested for its GIT stability using LMW fucoidan extracted from *F. vesiculosus* with M.W of 58.3 kDa and composed of 44% fucose and 26% sulfate [105]. These NPs showed antihyperglycemic, antioxidant, and anti-bacterial properties and a protective effect against degradation by GIT fluids after oral administration. The results of this study showed that upon the interaction between tea catechins and GIT aqueous fluids, the negative charges liberated from polyphenols interact with the positively charged chitosan. On the other hand, enhanced stability and high encapsulation efficiency were observed at acidic pH of the stomach conditions, which prevented gastric degradation of the components [105]. 

Fucoidan NPs prepared by the coacervation method showed higher anticoagulant activity than unprocessed fucoidan solution [106]. The results showed a twofold increase in activated partial thromboplastin time (aPTT) after fucoidan NPs administration compared to the unprocessed fucoidan solution which showed a 1.6-fold increase in aPTT [106]. 

Etman et al. investigated the anticancer effect of fucoidan nanoparticles (extracted from *Undaria Pinnatifida*, M.W= 47.9 kDa, and composition of sulfate content = 26%) against pancreatic cancer [107]. These NPs were prepared via polyelectrolyte complex. Ionic interaction between fucoidan and the positively charged, active targeting ligand lactoferrin (FUC/LF NPs) showed a biphasic release pattern in the form of initial burst release followed by a sustained release for 48 h. The results of the in vitro cytotoxicity study on pancreatic cancer cells showed that FUC/LF NPs possessed 2.3-fold lower IC_50_ compared to unprocessed fucoidan solution. This was attributed to enhanced cellular uptake by NPs through ligand-based endocytosis mediated by lactoferrin as a targeting for lactoferrin receptors and fucoidan as a targeting ligand for p-selectin receptors [107]. 

#### 3.5.3. Fucospheres 

A conventional way of processing fucoidan and chitosan polymers for the construction of microsphere-based drug delivery systems is by cross-linking fucoidan and chitosan to form “fucospheres” [108,109]. The drug loading and encapsulation efficiency inside fucospheres are mainly affected by the concentrations and molecular weights of fucoidan and chitosan, as well as drug properties [110]. 

Sezer et al. prepared bovine serum albumin (BSA)-loaded fucospheres composed of cross-linked chitosan and fucoidan with particle sizes ranging from 0.61 to 1.28 µm and smooth, poreless, spherical morphology [111]. The particle size depended on the concentration of fucoidan, chitosan, and BSA. The encapsulation efficiency of BSA varied between 51.8% and 89.5%. Increasing the concentration of fucoidan was directly correlated to the highest BSA encapsulation efficiency [111].

Fucospheres were used to improve the solubility and reduce the frequency of administration of the ofloxacin antibiotic using fucoidan (from *F. vesiculosus* with M.W = 80 kDa) cross-linked with chitosan [112]. Ofloxacin-loaded fucospheres showed an extended drug release over 8 h. The amount of ofloxacin released was decreased by increasing the concentration of fucoidan, and the overall drug release from fucospheres was slower than chitosan particles [112]. 

Fucospheres of the same origin and composition can also be applied topically for the treatment of dermal burns in vivo [113]. In this study, the formulation with the highest concentrations of fucoidan and chitosan showed the highest mucoadhesion, optimized surface charge, and particle size distribution. The macroscopical and histopathological examination revealed increased fibroblast migration, collagen accumulation, and accelerated re-epithelialization on days 7 and 14 of treatment by fucospheres [113]. 

#### 3.5.4. Scaffolds for Tissue Engineering

Fucoidan as a biomacromolecule can be used as a building block to produce self-assembling biomaterials, which can resemble the natural extracellular matrix necessary for cell culture and tissue engineering. Protein–polysaccharide hybrid hydrogels arranged via co-assembly or conjugation between peptides and polysaccharides provide a promising approach to tissue engineering [114]. This hybrid hydrogel can overcome the formerly reported problems such as lack of mechanical strength and low biological functionality associated with applying self-assembling polymers and synthetic peptides separately [114]. 

A thermodynamically driven hydrogel based on co-assembly between fucoidan and self-assembled peptide (SAP) was applied as a scaffold for skeletal muscle progenitor cells [115]. The myoblasts cultured on fucoidan scaffolds were smaller in size and had less multinucleated synthetia, with limited spreading and no observed toxicity. The scaffold matrix showed a 10-fold increase in stiffness compared to polysaccharide-free scaffolds [115]. 

Venkatesan et al. synthesized polymer-based scaffolds for bone tissue engineering, composed of chitosan, alginate, and fucoidan from *F. vesiculosus* [116]. The use of fucoidan was found to enhance the secretion of alkaline phosphatase (ALP), collagen type-1, osteopontin, and osteocalcin by human stem cells of adipose tissue compared to fucoidan-free chitosan–alginate scaffolds. Three-dimensional scaffolds composed of fucoidan and nano-hydroxylapatite uniformly dispersed in a chitosan matrix were prepared via a freeze-drying technique for bone tissue engineering. Fucoidan was selected to trigger the release of alkaline phosphatase (ALP), bone morphogenetic protein-2 (BMP-2), and osteocalcin, which is essential for the proliferation of osteoblasts. Additionally, fucoidan was reported to enhance collagen matrix formation and tissue angiogenesis [116].

## 4. Fucoidan Pharmacokinetics 

Pharmacokinetics (PK) generally describes the pathway of the drug into the body and how the body reacts to it in four main processes: absorption, distribution, metabolism, and elimination (ADME system). Comprehending the pharmacokinetic behavior of fucoidan is crucial for determining dosage recommendations and the most suitable dosage form for each condition to achieve effective therapeutic outcomes. 

The pharmacokinetic behavior of fucoidan from variable species was evaluated using experimental animals such as mice, rats, and rabbits after oral, topical, and parenteral administration. Recently, a group of researchers investigated the pharmacokinetic parameters of fucoidan extracted from *Laminaria japonica* after an intravenous injection of (50 mg/kg) in rabbits. The PK results showed a maximum plasma concentration (C_max_) of 110.53 µg/mL after a maximum time (T_max_) of 5 min [117,118]. In another study, IV injection of fluorescein-labeled fucoidan extracted from *F. vesiculosus* in mice showed C_max_ = 66.37 µg/mg and AUC = 198.11 µg/g·h [118,119].

Pozharitskaya et al. studied the pharmacokinetics and tissue distribution of an orally administered (100 mg/kg) dose of high-molecular-weight fucoidan (735 kDa) extracted from *F. vesiculosus* in rats. The results of this study showed a superior accumulation of fucoidan in the kidneys (C_max_ of 1.23 µg/g and a T_1/2_ of 7.26 h) compared to the spleen (C_max_ of 0.78 µg/g and a T_1/2_ of 9.32 h) and the liver (C_max_ of 0.53 µg/g and a T_1/2_ of 6.44 h). The findings of the previous studies demonstrated that the PK of fucoidan is strongly correlated with its molecular weight. Low-molecular-weight fucoidan was rapidly eliminated from blood after intravenous administration while high-molecular-weight fucoidan showed a prolonged circulation time of approximately 6.79 ± 1.63 h and high tissue distribution to filtration organs (i.e., kidneys, liver, and spleen) [120].

## 5. Scaling up Production of Fucoidan-Based Formulations: Possibilities and Challenges

Fucoidan generally and LMW specifically are associated with promising biological activity and high bioavailability. The molecular weight of algal extracts is remarkably influenced by the extraction and processing techniques. The pharmaceutical and nutraceutical processing of fucoidan extract is challenging due to the large molecular mass, viscous nature, and instability of some diverse components extracted from crude fucoidans. There are increasing concerns regarding extraction, depolymerization, and purification steps to maintain the integrity of the native structure and the purity of ingredients. The utilization of harsh extraction conditions such as acids, high temperatures, and long processing times should be avoided. Recently, novel delicate, eco-friendly processing technologies have been rapidly emerging, such as pressurized liquid extraction [121], ultrasound [122], microwave-assisted extraction [123], and biotechnology-mediated synthesis [124,125,126,127]. For instance, ultrasound-assisted extraction technique can positively affect the phytochemical composition of fucoidan (total fucoidan content and fucose/sulfate ratio) and it also accelerates the separation of the pharmacologically active compounds from the crude material, and hence, the anti-coagulant activity of fucoidan was improved after its topical administration to male Wistar rats [128].

Chauvierre et al. prepared GMP-grade ^99m^technetium–fucoidan as a diagnostic tool for cardiovascular diseases overexpressing the p-selectin receptor [129]. Firstly, the crude high-molecular-weight fucoidan was extracted from *Ascophyllum nodosum* and then depolymerized by non-selective oxidative reduction (ORD) followed by purification by ultrafiltration to obtain LMW fucoidan [129]. 

Regarding the large-scale production of fucoidan products, batch vials containing freeze-dried powder of fucoidan were obtained via the following steps: (1) dissolution of fucoidan in saline solution; (2) mixing; (3) 0.22 µm purification membrane filtration; (4) 0.22 µm sterilization membrane filtration; (5) aseptic filling and pre-stoppering; (6) freeze-drying (lyophilization); (7) final stoppering, capping, and compressing [129]. 

Production of oral fucoidan tablets was previously discussed in Section 3.1. The dried fucoidan extract can be mixed with excipients such as sodium croscarmellose, crospovidone, lactose monohydrate, and microcrystalline cellulose to improve its formulation parameters [55] (Table 2).

Despite all trials achieved by companies and researchers globally, scaling up algal products remains a challenging task. The optimum extraction method to achieve the highest degree of purification of fucoidan extract is crucial in scaling up the production of these products. In addition, there is a persistent need for a continuous supply of light, proper heat, nutrients, and hydrodynamic mixing to prevent settling and collapse. Nutrient source and supply are considered some of the most hampering obstructions as nutrients used at the laboratory scale are high in cost for mass production. On the other hand, the presence of nutrients produces impurities that need to be eliminated before proceeding with the scaling-up process [130,131]. 

**Table 2:** Rheological properties of different fucoidan forms.

	Pure Fucoidan Extract	Fucoidan-Based Hydrogel	Fucoidan/Buckwheat Starch Aqueous Paste
Apparent viscosity	Increased at high fucoidan concentration [132].	Fucoidan has a non-gelling nature, so viscosity is influenced by the addition of another gelling agent, i.e., carrageenan [32].	Increased at high fucoidan concentration [133].
Type of flow	-Non-Newtonian shear-thinning behavior at low shear rate (1–100 S^−1^). -A non-Newtonian shear-thickening behavior at high shear rate (100–1000 S^−1^) [132]. -A Newtonian flow behavior is seen with fucoidan solution extracted from *Fucus* *vesiculosus* at concentrations above 2% (*w/v*) [134].	Varies according to gelling agent and temperature (especially for thermo-responsive gelling agents) [135].	-At high a concentration: linear Newtonian flow. -At high a concentration: weak non-Newtonian shear-thinning pseudoplastic flow [133].
Factors affecting viscosity	-Algae species. -Molecular weight. -Degree of branching. The proportion of sulphates and uronic acids. -Temperature and pH. -Presence of ions and additional molecules [25].	Gelling agent concentration [136].	Fucoidan concentration [133].

## 6. Conclusions

The current research demonstrated the potential biopharmaceutical properties of fucoidan. In comparison with other polymers used, the incorporation of fucoidan into the pharmaceutical industry has resulted in the development of various smart delivery systems not only due to improved formulation properties, pharmacokinetic effects, and pharmacodynamic effects, but also thanks to fucoidan’s pharmacological activities. Considering these findings, more effective treatments have been developed for diseases such as lung cancer, fungal infections, and auto-immune disease, and for tissue engineering and other applications. The major obstacles to oral, topical, or parenteral fucoidan administration can be overcome by the diverse pharmaceutical formulation approaches based on the physicochemical properties of fucoidan. As we previously mentioned, the pharmacokinetic behavior of fucoidan is found to be strongly correlated with its molecular weight, as low-molecular-weight fucoidan was rapidly eliminated from circulation while high-molecular-weight fucoidan showed significantly prolonged circulation time. It is also noteworthy that LMW fucoidan is a prominent candidate for pharmaceutical formulation development and hence, recent research focuses on up-scaling production while retaining the native chemical backbone of fucoidan. As concluded in our review, differences in molecular weight, algal origin, and chemical composition have a great impact on the physicochemical and pharmacological properties as well as formulation techniques of fucoidan. The effect of these variables on fucoidan product outcomes leaves the door open for further research to assess their impact in the future. Moreover, further studies, particularly for engaging fucoidan in diagnostic and theranostic purposes, as well as well-established clinical trials to evaluate its safety and efficacy on humans, are also highly recommended in the future.

## Data Availability

Data can be provided by the authors on request.
